# SA4503 Mitigates Adriamycin-Induced Nephropathy via Sigma-1 Receptor in Animal and Cell-Based Models

**DOI:** 10.3390/ph18020172

**Published:** 2025-01-27

**Authors:** Hideaki Tagashira, Shinsuke Chida, Md. Shenuarin Bhuiyan, Kohji Fukunaga, Tomohiro Numata

**Affiliations:** 1 Department of Integrative Physiology, Graduate School of Medicine, Akita University, 1-1-1 Hondo, Akita 010-8543, Akita, Japan; 2 Bioscience Education and Research Support Center, Akita University, Akita 010-8543, Akita, Japan; schida@med.akita-u.ac.jp; 3 Department of Pathology and Translational Pathobiology, Louisiana State University Health Sciences Center-Shreveport, Shreveport, LA 71103, USA; shenu.bhuiyan@lsuhs.edu; 4 Department of Molecular and Cellular Physiology, Louisiana State University Health Sciences Center-Shreveport, Shreveport, LA 71103, USA; 5 Department of Pharmacology, Graduate School of Pharmaceutical Sciences, Tohoku University, Sendai 980-8578, Miyagi, Japan; kfukunaga@tohoku.ac.jp

**Keywords:** Sigma-1 receptor, SA4503, Adriamycin, nephrotic syndrome, podocyte injury

## Abstract

**Background/Objectives:** The Sigma-1 receptor (Sigmar1), an intracellular chaperone protein, is ubiquitously expressed throughout the body, but its role in peripheral organs, such as the kidneys, remains unclear. Here, we investigated the protective effects and molecular mechanisms of SA4503, a selective Sigmar1 agonist, on Adriamycin (ADR)-induced renal glomerular injury. **Methods:** Using in vitro and in vivo models, we evaluated the effects of SA4503 on ADR-induced podocyte injury, including podocyte survival, albumin permeability, urinary albumin levels, and Sigmar1-nephrin interactions. NE-100, a Sigmar1 antagonist, was co-administered to validate the specificity of the effects of SA4503. **Results:** Sigmar1 was highly expressed in podocytes and mouse kidney tissues. SA4503 significantly reduced ADR-induced podocyte injury and urinary albumin leakage in mice. Mechanistically, SA4503 preserved Sigmar1-nephrin interactions, which were disrupted in ADR-treated kidneys. This protective effect was abolished by NE-100 co-treatment, confirming the Sigmar1-dependency of SA4503’s action. **Conclusions:** These findings demonstrate that the activation of Sigmar1 by SA4503 protects against ADR-induced podocyte injury and glomerular damage, likely by stabilizing Sigmar1-nephrin interactions. Therefore, Sigmar1 represents a promising therapeutic target for glomerular diseases such as nephrotic syndrome.

## 1. Introduction

Nephrotic syndrome, such as focal segmental glomerulosclerosis (FSGS), is a common kidney disease caused by glomerular podocyte damage that ultimately leads to end-stage renal failure [1,2]. Clinical symptoms include massive proteinuria, hypoalbuminemia, and edema [2]. Although there are various theories about the pathogenesis of FSGS, including gene mutation, hypertension, and immune system abnormalities, the detailed pathological mechanism remains unclear [3]. In addition, current treatments have not achieved complete remission, so the development of new therapeutic drugs is required.

The sigma receptor (Sigmar) was first discovered as a subclass of opioid receptor and first cloned in 1996 [4,5]. There are two subtypes of sigma receptors, sigma-1 receptors (Sigmar1) and sigma-2 receptors (Sigmar2) [6,7]. Sigmar1 interacts with various proteins, such as IP_3_ receptors, and acts as a molecular chaperone protein that stabilizes functional expression by regulating ER-associated degradation (ERAD) [8,9,10]. Sigmar ligands are found in many existing drugs, and various compounds, such as 4-IBP, PRE-084, and SA4503 (Cutamesine), have also been synthesized [11,12]. Research on Sigmar1 ligands has made great progress in recent years, including the crystal structure of Sigmar1 and Sigmer-1 ligand-induced structural changes [13,14]. We have demonstrated that reduced functional expression of cardiac Sigmar1 leads to damage in cardiomyocytes [15]. Furthermore, the therapeutic potential of Sigmar1 agonists in the treatment of cardiovascular diseases has been highlighted [16,17,18]. Recently, it has been suggested that Sigmar1 may play a role in peripheral diseases beyond cardiovascular conditions, and attempts have been made to apply Sigmar1 ligands to peripheral diseases, such as cancer, infectious diseases, and respiratory diseases [19,20,21]. However, the potential utility of Sigmar1 ligands in treating glomerular diseases including retrospective studies of existing Sigmar1 agonist remains unclear and warrants further investigation.

In the present study, we confirmed the expression of Sigmar1 in renal glomerular podocytes using immunohistochemistry and RT-PCR. We investigated the effects of the selective Sigmar1 agonist SA4503 and the antagonist NE-100, which are highly specific Sigmar1 ligands [22,23], on podocyte function. Furthermore, we examined the protective effects of SA4503 in an ADR (also called doxorubicin)-induced FSGS-like nephropathy model, a well-established model of human glomerular injury characterized by podocyte damage and proteinuria. Our findings provide novel evidence that SA4503 preserves podocyte function and reduces ADR-induced injury, likely by stabilizing Sigmar1-nephrin interactions. These results identify Sigmar1 as a promising therapeutic target for glomerular diseases such as FSGS, and are important findings that serve as a foundation for future clinical trials.

## 2. Results

### 2.1. Sigmar1 Is Highly Expressed in Human and Mouse Glomerular Podocytes

**Figure 1:**
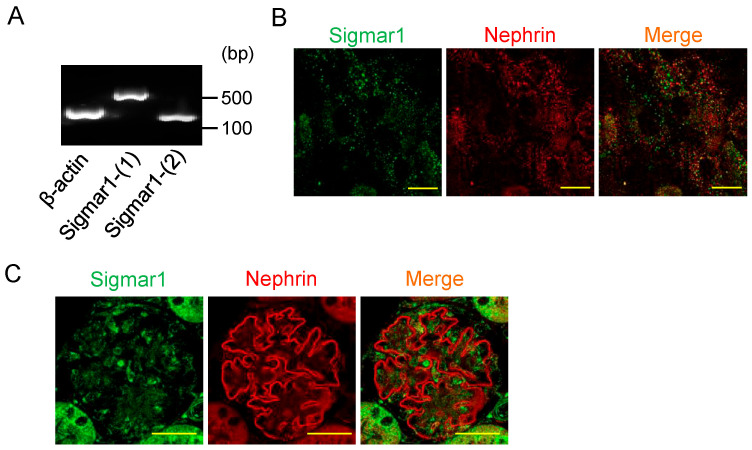
Sigmar1 is highly expressed in glomerular podocytes of human and mouse kidney tissues. (**A**) mRNA expression of *Sigmar1* and *β-actin* gene. PCR products were obtained from a human podocyte cell line. (**B**) Immunofluorescence staining of sigmer 1 (green) and nephrin (red, a podocyte marker) in cultured podocytes. Scale bar = 20 µm. (**C**) Representative photographs of immunohistochemical staining for Sigmar1 (green) and nephrin (red, podocyte marker) in mouse kidney. Scale bar = 20 µm.

We investigated the expression of *Sigmar1* mRNA levels in renal glomerular podocytes using a human podocyte cell line. The results of RT-PCR using two types of *Sigmar1* primers confirmed that human podocytes expressed a large amount of *Sigmar1* (Figure 1A). In addition, the expression of Sigmar1 and nephrin, a well-established podocyte marker, at the protein level was detected by immunocytochemical staining (Figure 1B). Furthermore, immunohistochemical analysis of mouse renal tissue revealed that Sigmar1 is expressed in glomerular podocytes and is in close proximity to nephrin in the slit diaphragm region (Figure 1C). This finding suggests that Sigmar1 may play a role in podocyte-specific structures essential for maintaining glomerular function.

### 2.2. Sigmar1 Agonist SA4503 Ameliorates ADR-Induced Podocyte Injury in Human Podocytes

**Figure 2:**
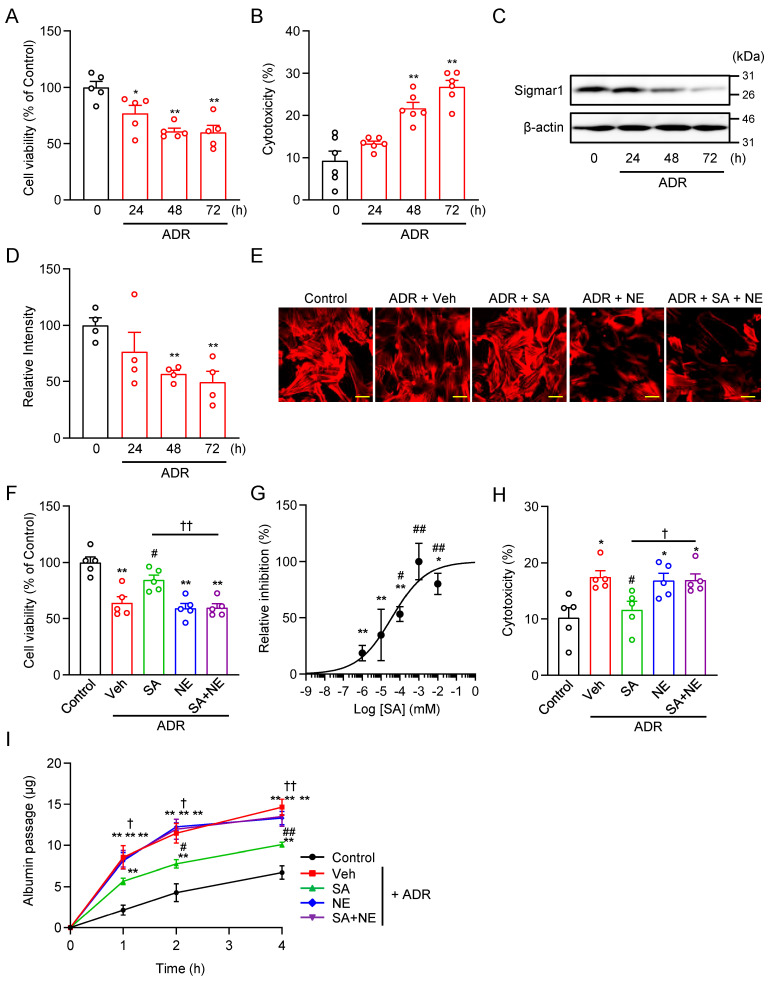
Sigmar1 activation by SA4503 ameliorates ADR-induced podocyte injury. (**A**,**B**) Time course of ADR (0.3 µg/mL)-induced reduction in cell viability (**A**) (*n* = 5) and cytotoxicity (**B**) (*n* = 6) in human podocytes. The cell viability was presented as a relative value (%) of the absorbance obtained in each experiment, normalized to the average value of the control condition. Cell injury in podocytes occurs up to 48 h after treatment with ADR. (**C**) Representative images of Western blot analysis of Sigmar1 and β-actin. (**D**) Densitometric quantification of Sigmar1/β-actin immunoreactive bands (*n* = 4). (**E**) Representative images of podoocytes stained with rhodamine-conjugated phalloidin for each experimental group. Scale bar = 20 µm. (**F**) The MTT assay assessed cell viability of ADR-treated human podocytes with or without SA4503 (SA) (1 µM) and/or NE-100 (NE) (1 µM) (*n* = 5). (**G**) Statistical analysis of the dose-dependent inhibition curve of SA4503 against ADR-induced reduction in cell viability (*n* = 5). (**H**) Cytotoxicity was evaluated through the LDH release assay (*n* = 5). (**I**) In vitro permeability of albumin through podocyte monolayers in each experimental group (*n* = 6–8). The passage of albumin was determined at the indicated hours after albumin administration. Each column represents the mean ± S.E.M. *, *p* < 0.05 and **, *p* < 0.01 indicate a significant difference versus control cells. #, *p* < 0.05 and ##, *p* < 0.01 indicate a significant difference versus ADR + vehicle (Veh) treated cells. †, *p* < 0.05 and ††, *p* < 0.01 indicate a significant difference versus ADR + SA4503 (SA) treated cells.

We next investigated the effect of Sigmar1 ligand on renal glomerular podocytes. It is well known that 0.3 µg/mL ADR induces podocyte damage in human podocytes [24]. To determine the time course of ADR-induced cytotoxicity, we assessed cell viability using an MTT assay and measured cell damage via LDH release. A significant decrease in cell viability and cell damage was observed at 48 h (Figure 2A,B). Western blot analysis indicates a decrease in Sigmar1 expression at 24 h, with a significant reduction observed at 48 h (Figure 2C,D). Based on these observations, we selected 0.3 µg/mL ADR treatment for 48 h as the optimal condition for subsequent experiments to evaluate the protective effects of SA4503. These results indicate that ADR-induced podocyte injury is associated with a time-dependent reduction in Sigmar1 expression, suggesting a potential link between Sigmar1 levels and podocyte survival.

Previously, it was reported that Sigmar1 agonists exhibit ameliorative effects on pathological cellular conditions following chronic, but not acute, treatment, likely through stabilization of cellular homeostasis [18]. Based on this, SA4503 treatment was performed for 48 h in this study. Treatment with SA4503 dose-dependently improved ADR-induced damage, as evidenced by the restoration of cell morphology and increased cell viability (Figure 2E–G). The 50% inhibition concentration (IC_50_) was determined to be 37 nM with a Hill slope of 0.5 (Figure 2G). During these studies, no cytotoxicity of SA4503 in podocytes was observed. Based on the dose-response analysis, we selected a concentration of 1 μM SA4503 to further evaluate its protective effects at 48 h after ADR application. SA4503 treatment also suppressed ADR-induced cytotoxicity (Figure 2H). On the other hand, treatment with 1 μM NE-100, a selective Sigmar1 antagonist [22], did not alter podocyte damage. Notably, co-treatment with NE-100 abolished the protective effects of SA4503, indicating that the observed cytoprotective effects are Sigmar1-dependent (Figure 2E,F,H).

Furthermore, to examine the barrier function of podocytes, we performed an in vitro albumin permeability assay, a well-established method to evaluate podocyte function [24,25,26]. Consistent with previous studies [24], ADR treatment for 48 h resulted in a significant increase in the in vitro passage of albumin across the podocyte monolayer (Figure 2I). SA4503 treatment (1 μM) significantly reduced ADR-induced albumin permeability to near control levels. However, this improvement was abolished by co-treatment with NE-100 (1 μM), a selective Sigmar1 antagonist, confirming that the effect of SA4503 is Sigmar1-dependent (Figure 2I).

**Figure 3:**
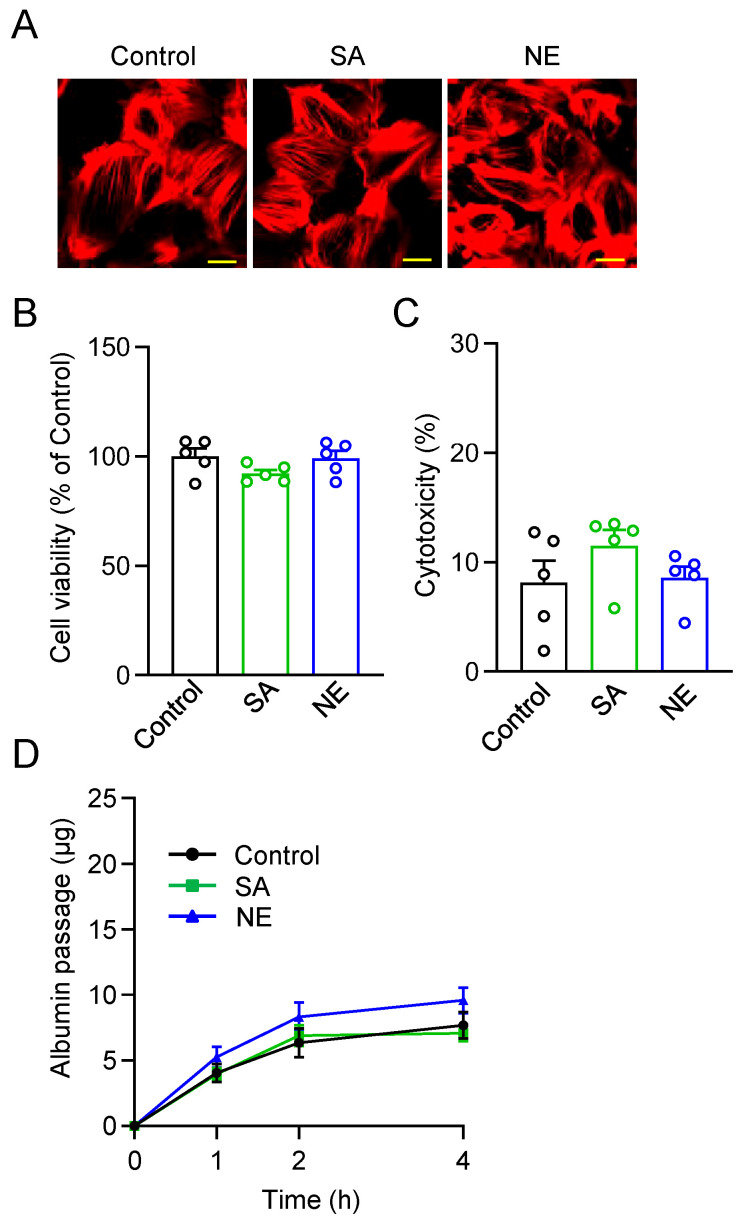
SA4503 and NE100 have no effect on intact human podocytes. (**A**) Representative images of podocytes stained with rhodamine/phalloidin for each experimental group. Scale bar = 20 µm. (**B**,**C**) Cell viability (**B**) (*n* = 5) and cytotoxicity (**C**) (*n* = 5) of intact human podocytes treated with SA4503 (SA) (1 µM) or NE-100 (NE) (1 µM). The cell viability was presented as a relative value (%) of the absorbance obtained in each experiment, normalized to the average value of the control condition. (**D**) In vitro permeability of albumin through podocyte monolayers in SA4503 or NE-100 treated human podocytes (*n* = 9). The albumin passage was measured at the indicated hours (h) after albumin administration. Each column represents the mean ± S.E.M.

In contrast, treatment with SA4503 or NE-100 alone in intact, untreated podocytes did not affect cell morphology, cell damage, or albumin permeability, indicating no adverse effects or functional changes under basal conditions (Figure 3A–D).

### 2.3. Glomerular Sigmar1 Expression Was Decreased in ADR-Induced Glomerular Injury Mice

**Figure 4:**
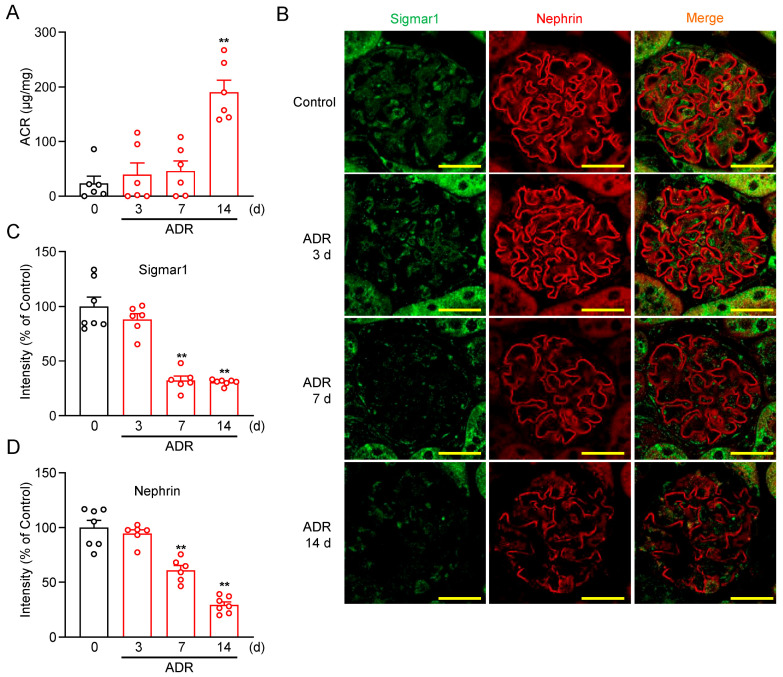
Time course of glomerular injury and sigmar1 expression in ADR-treated mice. (**A**) Time course of urinary albumin/creatinine ratio (ACR) in mice 3, 7, and 14 days (d) after ADR injection (18 mg/kg) (*n* = 6). (**B**) Immunofluorescence staining demonstrates the abundance and distribution pattern of Sigmar1 (green) and nephrin (red) in different time as indicated. Scale bar = 20 µm. (**C**,**D**) Quantification of the expression level of Sigmar1 (**C**) and nephrin (**D**) by measuring fluorescence intensity (*n* = 6–7). Each column represents the mean ± S.E.M. **, *p* < 0.01 indicates a significant difference versus control mice.

To verify the in vivo effects of Sigmar1 ligands, we performed a study using a mouse model of ADR-induced glomerular injury. ADR (18 mg/kg, i.v.) was administered to C57BL/6J mice following established protocols [24,27]. Compared to control mice, significant urinary albumin leakage was observed starting on 14 days (d) after the ADR injection (Figure 4A).

Previous studies have shown that ADR-induced reductions in nephrin expression disrupt the slit diaphragm, leading to urinary albumin leakage [24,27,28]. We next analyzed the expression levels of Sigmar1 and nephrin in glomeruli using immunohistochemistry. Interestingly, Sigmar1 and nephrin expression levels were both reduced 7 d post-ADR injection, preceding the onset of albuminuria (Figure 4B–D). Notably, the decrease in Sigmar1 expression was more pronounced than that of nephrin (Figure 4B–D). These results suggest that ADR-induced podocyte dysfunction and subsequent albuminuria may be mediated, at least in part, by an early and significant decrease in Sigmar1 expression, which likely contributes to the downregulation of nephrin and the loss of glomerular barrier integrity.

### 2.4. Sigmar1 Activation by SA4503 Ameliorates ADR-Induced Glomerular Injury in Mice

**Figure 5:**
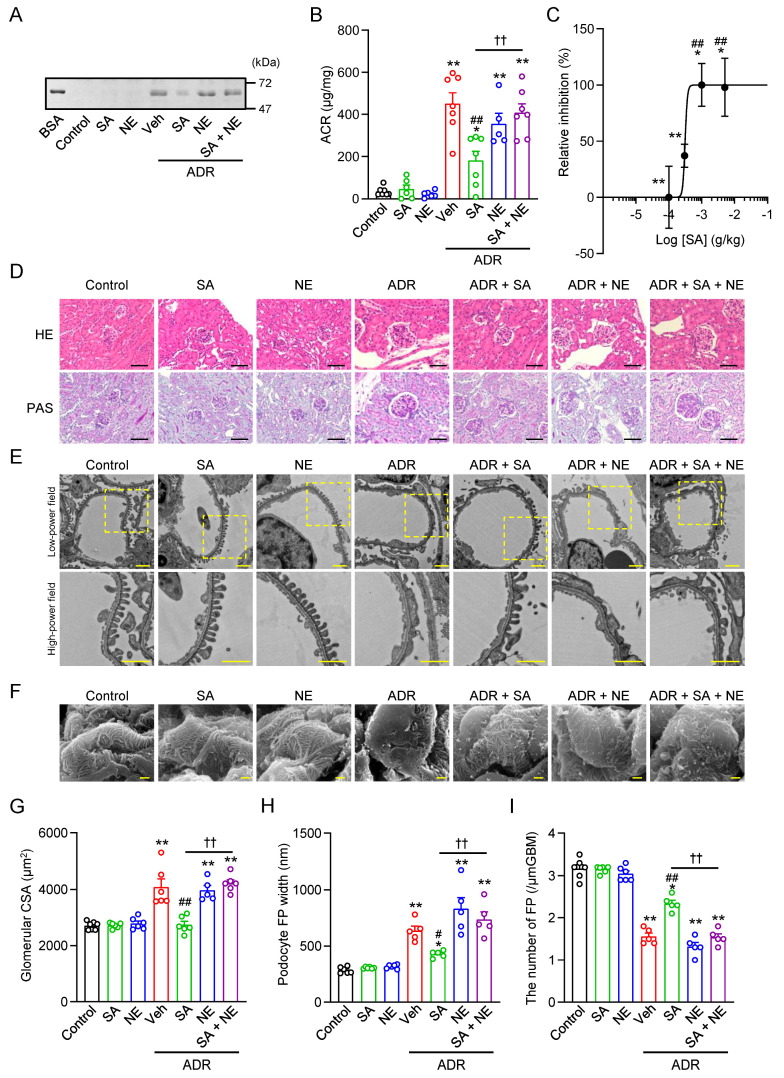
Activation of glomerular Sigmar1 by SA4503 ameliorates glomerular injury in ADR-treated mice. (**A**) Representative SDS-PAGE shows the urine proteins in mice treated with or without ADR (18 mg/kg), SA4503 (SA) (1 mg/kg), and/or NE-100 (NE) (1 mg/kg) as indicated. BSA (0.1 µg/µL) is shown as a positive control. (**B**) The urinary albumin/creatinine ratio (ACR) in mice of each treatment (*n* = 5–7). (**C**) Statistical analysis of the dose-dependent inhibition curve of SA4503 (SA) against ADR-induced excretion of albuminuria (*n* = 5–7). (**D**) Renal morphological analysis in mice treated with or without ADR, SA and/or NE (HE staining and PAS staining) (*n* = 5–6). (**E**) Microstructure of podocytes from mice treated with or without ADR, SA and/or NE by transmission electron microscopy. (**F**) Scanning electron micrographs of podocyte foot processes (FP) in mice treated with or without ADR, SA and/or NE. (**G**) Evaluation of the glomerular cross-sectional area (CSA) of each experimental group, based on multiple observations as depicted in (**D**) (*n* = 5–6). (H, I) Measurement of FP width (**H**), and the number of FP per area of the glomerular basement membrane (GBM) (**I**) of each experimental group, based on multiple observations as depicted in (**E**) (*n* = 5–6). Each column represents the mean ± S.E.M. *, *p* < 0.05 and **, *p* < 0.01 indicate a significant difference versus control mice. #, *p* < 0.05 and ##, *p* < 0.01 indicate a significant difference versus ADR + vehicle (Veh) treated mice. ††, *p* < 0.01 indicate a significant difference versus ADR + SA4503 (SA) treated mice.

Using a mouse model of glomerular injury 14 d after ADR injection, with the characteristics shown in Figure 4A, the effects of Sigmar1 ligands on renal glomerular injury were examined. Treatment with SA4503 dose-dependently suppressed ADR-induced albumin leakage, with an IC_50_ value of 308 µg/kg as determined by nonlinear regression analysis (Figure 5A–C). During these studies, no side effects of SA4503 were detected, as evidenced by body weight changes, behavior, respiratory observations, and kidney histology analysis. In contrast, treatment with SA4503 or NE-100 alone in control mice did not alter urinary albumin levels, confirming the lack of effect under basal conditions (Figure 5A,B). Co-treatment with NE-100 (1 mg/kg), a selective Sigmar1 antagonist, abolished the protective effects of SA4503 on ADR-induced albuminuria, indicating that the effects of SA4503 are mediated through Sigmar1 activation (Figure 5A,B).

Morphometric analysis of kidney histology revealed an increase in glomerular volume in ADR-treated mice kidneys compared to control mice kidneys (Figure 5D,G). However, the increase in glomerular volume was ameliorated by SA4503 treatment (Figure 5D,G). In addition, administration of NE-100 alone did not induce glomerular hypertrophy, but co-administration of NE-100 abolished the effect of SA4503 (Figure 5D,G). Furthermore, since SA4503 ameliorated ADR-induced increased albuminuria, we hypothesized that it might also prevent podocyte damage and loss. Foot process (FP) effacement has long been known to be useful in diagnosing nephrotic syndrome in rodents and humans [29,30]. To evaluate podocyte morphology, kidney sections were examined using transmission electron microscopy (TEM) at 2000× to 5000× magnification. ADR treatment significantly increased FP effacement, as evidenced by broader FP widths compared to control mice (Figure 5E,H,I). Notably, SA4503 treatment significantly reduced FP effacement by approximately 60%, although podocyte morphology did not fully recover to control levels. Co-administration of NE-100 negated the effect of SA4503 in improving podocyte effacement (Figure 5E,H,I). Administration of SA4503 or NE-100 alone for control mice did not change podocyte morphology, FP width, or podocyte number (Figure 5E,H,I). Consistent with these short-term study, no toxicity was reported in SA4503 administration for 42 days in L4-L5 rhizotomy mice model and 28 days in clinical trials (Long-term study) [31,32].

### 2.5. Sigmar1 and Nephrin Form a Complex, and SA4503 Suppresses the Decrease in Sigmar1 and Nephrin in ADR Nephropathy In Vivo

**Figure 6:**
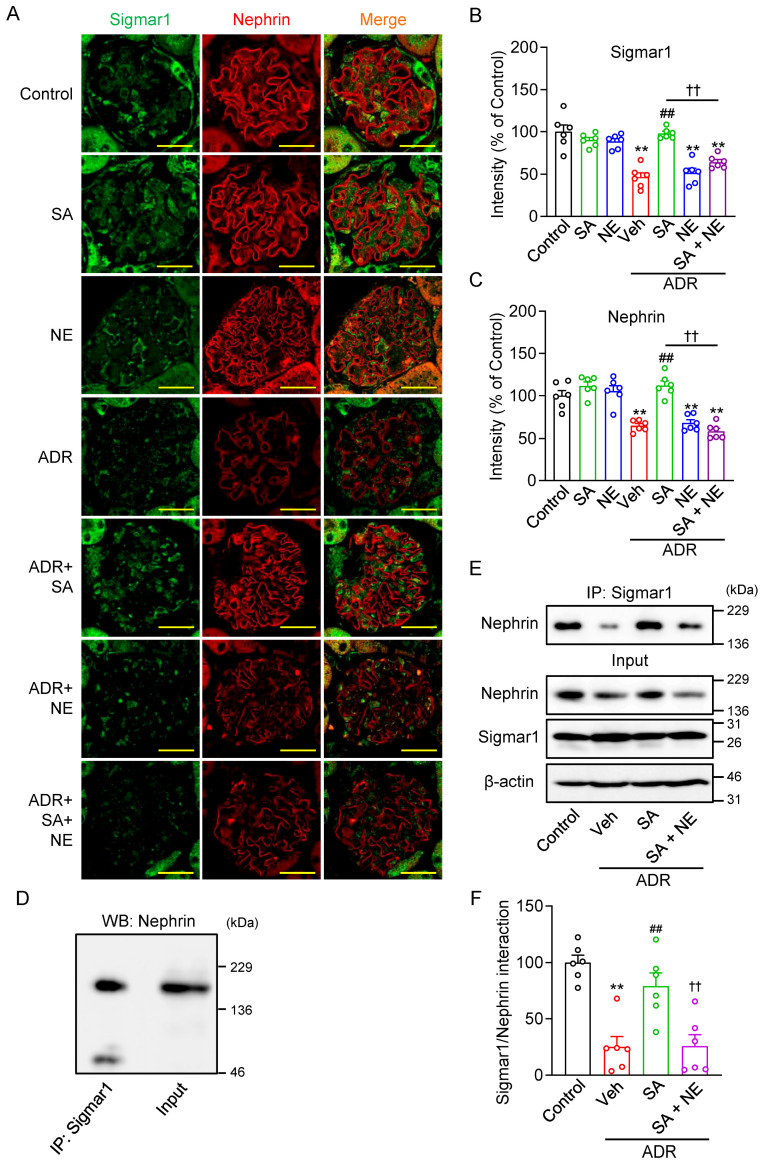
The interaction between Sigmar1 and nephrin in mouse renal tissues. (**A**) Immunofluorescence image of renal tissues from mice treated with or without ADR (18 mg/kg), SA4503 (SA) (1 mg/kg), and/or NE-100 (NE) (1 mg/kg), showing Sigmar1 in green and nephrin in red. Scale bar = 20 μm. (**B**,**C**) Quantification of the expression levels of Sigmar1 (**C**) and nephrin (**D**) by measuring fluorescence intensity (*n* = 6–7). (**D**) Immunoprecipitation (IP) of Sigmar1 from mouse kidney lysate. The same amounts of proteins from the lysate were used for IP. (**E**) IP of Sigmar1 from kidney lysate of mice treated with or without ADR, SA and/or NE. After immunoprecipitation with the anti-Sigmar1 antibody, Western blot analysis of the IP sample was performed using anti-nephrin antibody (upper panel). Western blot analysis of kidney extracts (lysate) was performed using anti-nephrin, Sigmar1, and β-actin antibodies (lower panel). (**F**) Densitometric quantification of immunoreactive bands of nephrin immunoprecipitated by Sigmar1 in mouse kidney. Data are expressed as percentages of the value for control mice (*n* = 6). Each column represents the mean ± S.E.M. **, *p* < 0.01 indicates a significant difference versus control mice. ##, *p* < 0.01 indicates a significant difference versus ADR + vehicle (Veh) treated mice. ††, *p* < 0.01 indicates a significant difference versus ADR + SA4503 (SA) treated mice.

To elucidate the mechanism by which SA4503 improves ADR-induced glomerular injury, we analyzed the expression levels of Sigmar1 and nephrin by immunohistochemistry in mouse kidneys 14 d after ADR, SA4503, and/or NE-100 treatment. Immunohistochemistry revealed that ADR significantly reduced glomerular Sigmar1 and nephrin expression levels, which were restored by SA4503 treatment. Co-administration of NE-100 abolished this effect, confirming Sigmar1-dependent protection (Figure 6A–C).

The immunohistochemical staining images in Figure 1C, Figure 4B and Figure 6A showed Sigmar1 and nephrin in close proximity within glomeruli, suggesting potential complex formation. This hypothesis was confirmed by immunoprecipitation (IP) assays, where nephrin co-precipitated with Sigmar1 in vivo (Figure 6D). Furthermore, by quantifying the intensity of nephrin immunoreactivity between IP samples and kidney tissue lysates, we found that the proportion of nephrin that interacted with sigmer-1 was 65.8% of total nephrin (Figure 6D). Interestingly, ADR treatment reduced Sigmar1-nephrin complex formation to 25 ± 9% of control levels, which was more pronounced than the overall decrease in nephrin expression. SA4503 treatment restored the Sigmar1-nephrin interaction to 79 ± 12% of control levels. However, co-administration of NE-100 negated this recovery, further supporting the role of Sigmar1 in complex stabilization (Figure 6E,F). These results indicate that SA4503 exerts its protective effects by promoting Sigmar1-nephrin complex formation, which may prevent nephrin degradation and maintain glomerular structural integrity under ADR-induced injury.

## 3. Discussion

The effects of Sigmar1 ligands on the kidney, especially glomerular podocyte disease, have not been fully elucidated, despite the progress of research into their application to pathological conditions. In the present study, we demonstrated for the first time that Sigmar1 is abundant in glomerular podocytes. Additionally, we found that the Sigmar1-selective agonist SA4503 prevents ADR-induced podocyte injury in vitro. Furthermore, SA4503 ameliorates ADR-induced renal glomerular injury in vivo. Although the mechanism of action is not fully elucidated, our findings suggest that these effects may be mediated, at least in part, by the inhibition of nephrin downregulation through the promotion of complex formation of Sigmar1 with nephrin via Sigmar1 activation.

Sigmar1 is highly homologous among rats, mice, and humans [33]. Our previous study suggested that Sigmar1 is expressed in rodent renal tissue, and that Sigmar1 down-regulation is involved in pressure overload-induced renal hypertrophy [34]. Subsequently, Hosszu et al. reported the Sigmar1 expression in renal tubules and the usefulness of Sigmar1 agonists for renal ischemia-reperfusion injury [35]. In addition, other groups have found that PRE-084, another Sigmar1 selective agonist, improves adenine-induced renal injury through increased Sigmar1 expression in renal tubules [36]. However, the expression of Sigmar1 in the renal glomerulus has not been elucidated. In this study, we proved the expression of Sigmar1 not only in renal tubules but also in glomeruli using human podocyte cell lines and mouse renal tissues (Figure 1). Furthermore, the results of the Western blot in Figure 2C show that Sigmar1 was expressed at higher levels in podocytes and was reduced in correlation with ADR-induced injury. Additionally, functional expression of Sigmar1 and nephrin in mice decreased 7 d before albuminuria was detected in the glomeruli, and the decrease in Sigmar1 expression was more pronounced than that of nephrin (Figure 4). These results demonstrate that Sigmar1 downregulation is involved in podocyte pathology. However, the mechanism of Sigmar1 downregulation in podocytes remains unclear, and further elucidation of the detailed mechanism is required in future studies.

Sigmar ligands are known to exist in many drugs, and many have been synthesized [11,12]. Among these, SA4503 and NE-100 are highly potent and selective Sigmar1 ligands [11,37]. SA4503 was synthesized as a selective Sigmar1 agonist in 1996 [23]. Matsuno et al. reported that SA4503 showed high affinity for the [^3^H](+)-pentazocine-labeled Sigmar1 subtype, with an IC_50_ of 17.4 ± 1.9 nM, but its affinity for the Sigmar2 subtype was approximately 100-fold lower, with little affinity for 36 other receptors, ion channels, and second messenger systems [23]. Subsequent studies have demonstrated that SA4503 has protective effects against cardiovascular disease, depression, and stroke [21,38]. Meanwhile, NE-100 was synthesized by Chaki et al. as a potent and selective Sigmar1 antagonist in 1994 [22]. They reported that NE-100 inhibited [3H](+)-pentazocine binding to Sigmar1 binding sites, with an IC_50_ of 1.54 ± 0.26 nM [22]. NE-100 was widely used to demonstrate the Sigmar1-mediated effects of Sigmar1 agonists [39,40,41]. In the present study, we investigated the effects of Sigmar1 ligands on renal glomerular podocytes. There was no effect when 1 μM (for podocyte) or 1 mg/kg (for mice) of SA4503 or NE-100 was treated alone, suggesting that these concentrations of Sigmar1 ligands do not have endogenous Sigmar1 inhibitory activity or cytotoxicity (Figure 3 and Figure 5). An important finding of this study was that ADR-induced glomerular injury and albuminuria were improved by SA4503 treatment in vitro and in vivo, whereas the ameliorative effect of SA4503 treatment was negated by co-treatment with NE-100 (Figure 2 and Figure 5). These findings suggest that SA4503 activates Sigmar1 in the renal glomerulus and exerts a protective effect on podocytes. However, this study focuses on the effect of SA4503 on ADR-induced FSGS-like glomerular disease, but it does not completely mimic the inflammation and fibrosis observed in diabetic nephropathy and membranous glomerulopathy. To gain a more comprehensive understanding of the role of Sigmar1 in various glomerular pathologies, it will be essential to investigate the effects of SA4503 on other glomerular pathology models or to examine the pathological susceptibility in Sigmar1-deficient mice in the future.

Glomerular hypertrophy is an important diagnosis for glomerular diseases [42,43]. In the present study, SA4503 treatment significantly inhibited ADR-induced glomerular hypertrophy (Figure 5B,C,F). Furthermore, to examine the morphology of renal glomerular podocytes in detail, renal tissue was observed using an electron microscope. FP effacement has long been known to be useful for diagnosing proteinuric renal diseases in humans as well as in animal models, and it has a high correlation with the pathology [29,30]. In the ADR-treated group, FP width was significantly increased and the number of podocytes was significantly decreased, confirming FP effacement (Figure 5D,G,H). On the other hand, SA4503 treatment partially, but significantly, prevented these disorders (Figure 5D,G,H). These results and verification using a podocyte cell line suggest that SA4503 acts on podocyte Sigmar1 and suppresses ADR-induced FP effacement, thereby suppressing albumin leakage.

One of the most important findings of this study is that the interaction between Sigmar1 and nephrin was confirmed by IP assay. In the renal tissue of ADR mice, the complex formation was significantly reduced, even more than the reduction in nephrin expression (Figure 6). Accumulating evidence suggests that ADR induces a decrease in nephrin expression, which is closely associated with urinary albumin leakage [24,27,28]. Nephrin forms a complex with podocin and other proteins, and it plays an important role as a component of the slit diaphragm [1,44]. In addition, nephrin gene (*NPHS1*, *NPHS2*) mutations are known to be causative genes of FSGS, a representative disease of nephrotic syndrome [45]. Indeed, NPHS1 homozygous knockout mice die shortly after birth due to massive albuminuria caused by slit diaphragm deficiency [46]. Additionally, mice with inducible RNA interference-mediated nephrin knockdown developed mild proteinuria, FP effacement, and podocyte apoptosis [47]. Furthermore, decreased expression and redistribution of nephrin in podocytes of patients with primary acquired nephrotic syndrome have been reported as a potential mechanism for proteinuria [48]. Sigmar1 is a molecular chaperone protein that interacts with various molecules [10,12,49,50], and it is a unique receptor protein that protects the partner protein from degradation via the ERAD system by forming a complex, thereby stabilizing its functional expression [8,9]. ERAD has attracted attention as a function responsible for protein quality control in the kidney, and it is closely related to ER stress signaling such as transcription activation factor 6 (ATF6) and protein kinase R-like ER kinase (PERK) [51,52]. Importantly, accumulating evidence indicates that nephrin is degraded via the ERAD pathway [53,54]. For instance, ERAD inhibition led to reduced nephrin expression and exacerbated albuminuria in diabetic mice [55]. Summarizing the results of this study and previous reports, Sigmar1 may prevent FP effacement by inhibiting the disassembly of the slit membrane protein complex through interaction with nephrin. However, the detailed mechanism of pathogenesis via Sigmar1 downregulation remains unknown; therefore, further investigation, especially ER stress (such as ATF6 and PERK) and ERAD signaling, using Sigmar1 knockout mice or siRNA knockdown podocytes, is required. In addition, since the reduction in ER stress [36] and the activation of Akt-mediated nitric oxide signaling [35] by Sigmar1 agonists have been reported in renal tissue, this possibility should also be taken into consideration. Taken together, the glomerular protective effects of SA4503 may be mediated by suppressing ER stress and anti-inflammatory effects via Sigmar1 activation, as well as by maintaining the formation of the Sigmar1-nephrin complex and inhibiting nephrin downregulation directly or indirectly. On the other hand, although the ADR-induced glomerular disease model used in this study shows a human FSGS-like phenotype, it does not completely mimic the inflammation and fibrosis observed in diabetic nephropathy and membranous glomerulopathy. Future studies are needed to clarify the detailed mechanism of Sigmar1-mediated glomerular protection and to verify the mechanism in other animal models.

SA4503 has been in Phase II trials for human stroke [32]. As a result, it was reported that no adverse side effects were observed after 28 days of treatment and 56 days of follow-up [32]. However, no trials have been conducted on its effect on renal glomerular disease, and its effect remains unknown. In this study, the effect of SA4503 was examined using ADR glomerular nephropathy mice model, and its usefulness was demonstrated. These results provide the basis for translational research, although further validation in other animal models, such as diabetic nephropathy and membranous glomerulopathy, is required. In addition, clinical examination of genetic variability (such as species, racial differences, polymorphism, and mutation), pharmacokinetic aspects (drug tissue accumulation, absorption, distribution, metabolism, and excretion), and Sigmar1 selectivity is also necessary, and clinical trials on the effects of SA4503 on nephrotic syndrome are expected to be conducted in the future.

## 4. Materials and Methods

### 4.1. Materials

The following regent were used in this study: SA4503 (Cutamesine) (Sigma-Aldrich, St. Louis, MO, USA) and NE-100 (Cayman Chemical, Ann Arbor, MI, USA), and dissolved in water. Adriamycin (ADR) was purchased from Fujifilm Wako (Osaka, Japan). Rhodamine-conjugated phalloidin were obtained from Abcam (ab235138, Cambridge, UK).

### 4.2. Animals

All animal experiment were performed in strict accordance with the Guide for Care and Use of Laboratory Animals and received prior approval from the Animal Ethics Committee of Akita University (Akita, Japan). We used 8–10 weeks old male C57BL/6J mice that were acclimated to the laboratory environment. Mice were housed under controlled environmental conditions, which incubated a temperature of 25 ± 1 °C, humidity regulation of 50–60%, and a 12 h light/dark schedule. Mice had ad libitum access to standard rodent chow and water. Health conditions were monitored daily throughout the experimental period, and appropriate care was provided as necessary to ensure animal welfare.

### 4.3. ADR Injection and Drug Administration in Mice

Mice in the ADR group were treated with intravenous administration of ADR (18 mg/kg, Fujifilm Wako, Osaka, Japan) via tail vein injection, following established protocols for ADR-induced glomerular injury in C57BL/6J mice [24,27]. C57BL/6J mice were selected due to their partial resistance to ADR, which allows for the controlled induction of nephropathy without excessive systemic toxicity. Spot urine samples were collected at 3, 7, and 14 days (d) post-ADR injection. Renal tissues were surgically removed under isoflurane anesthesia, snap-frozen in liquid nitrogen for protein and gene analysis, or fixed in 4% paraformaldehyde for histological examination. Vehicle (0.9% saline), SA4503 (0.1–5 mg/kg/day, i.p.), and NE-100 (1 mg/kg/day, i.p.) were administered intraperitoneally once daily for 14 consecutive days, starting immediately after ADR injection. The injection volume was standardized at 1 mL/100 g body weight for all treatments. The dose ranges were selected based on previous studies demonstrating their efficacy and safety in rodent models [56,57].

### 4.4. Western Blot Analysis

Western blot analysis followed established procedures, as previously described [58,59]. Immunoprecipitation (IP) assay was performed using the Capturem™ IP & CO-IP kit (Takara-Bio, Otsu, Japan) according to the manufacturer’s protocol. Whole-cell lysates and IP samples were resolved by SDS-PAGE using 10% or 13.5% polyacrylamide gels. Proteins were then transferred to polyvinylidene difluoride (PVDF) membranes and incubated with primary antibodies, specifically anti-nephrin antibody (1:500) (SC-376522, Santa Cruz, CA, USA), anti-Sigmar1 antibody (1:100 for IP, 1:500 for Western blot) (15168-1-AP, Proteintech, Rosemont, IL, USA), and anti-β-actin antibody (1:2000) (A1978, Sigma-Aldrich, St. Louis, MO, USA) as internal standard. After them, the blots were incubated with the secondary antibody of mouse IgG (1:2000) (NA9310, Amersham, Little Chalfont, UK) or rabbit IgG (1:2000) (NA9340, Amersham, Little Chalfont, UK). The PVDF membrane was visualized using a chemiluminescence reagent (ImmunoStar Zeta, Fujifilm Wako, Osaka, Japan) and detected using a LumiVision Pro 400EX system (Aisin Seiki Co., Ltd., Aichi, Japan). Quantitative analysis was performed using the ImageJ program (version 1.54m, NIH).

### 4.5. Quantitative Analysis of Albuminuria and Protein/Creatinine Concentration

Spot urine samples were centrifuged at 3000 g for 10 min at 4 °C, and the supernatants were stored at −20 °C until analysis. Urinary albumin concentrations in spot urine were quantified by separation by 10% SDS-polyacrylamide gel electrophoresis followed by Coomassie brilliant blue (CBB) staining according to the manufacturer’s protocol (Fujifilm Wako, Osaka, Japan). Concentration was analyzed by ChemiDoc Touch Imaging System (Bio-Rad, Hercules, CA, USA) and the data were normalized with BSA standard (0.1, 0.5, 1 µg/µL). Urine creatinine levels were quantified in the same samples by commercial kits (Fujifilm Wako, Osaka, Japan). The amount of leaked albumin was normalized with urinary creatinine concentration. Absorbance at 570 nm was recorded by Infinite M200 microplate reader (Tecan Group Ltd., Männedorf, Switzerland).

### 4.6. Mouse Tissue Collection and Histochemical Staining

After deep anesthesia with ketamine (100 mg/kg, i.p., Daiichi Sankyo Pharmaceutical Co., Ltd., Tokyo, Japan) and xylazine (10 mg/kg, i.p., Bayer Yakuhin, Ltd., Osaka, Japan), tissue collection and perfusion fixation were performed. Anesthesia depth was confirmed by the absence of a pedal withdrawal reflex. Mice were then perfused via the left ventricle with phosphate-buffered saline (PBS), followed by 4% paraformaldehyde phosphate buffer solution (PFA) (Nacalai Tesque, Kyoto, Japan), at controlled flow rate. The kidneys were then fixed in 4% PFA (4 °C, overnight) and subsequently embedded in paraffin. Tissue sections of 3 μm thickness were cut from the paraffin blocks and stained with H&E (HE: hematoxylin and eosin) and periodic acid Schiff (PAS) according to standard protocols. The slides were imaged using a BZ-X800 Keyence inverted microscope (Osaka, Japan). Kidney sections from a minimum of five mice per group were analyzed. For immunohistochemical staining, the specimens were incubated with primary antibodies, specifically anti-nephrin antibody (1:200) (SC-376522, Santa Cruz, CA, USA) and anti-Sigmar1 antibody (1:200) (15168-1-AP, Proteintech, IL, USA) in PBS (1% BSA) at 4 °C for overnight, after they were deparaffinized with Histofine solution (Nitirei, Tokyo, Japan). The glomerular tuft cross-sectional area and fluorescence intensity were quantified by a computer image analysis system (Image J, version 1.54m, NIH).

### 4.7. Electron Microscopy

For transmission electron microscopy (TEM), kidneys were perfused with 3% glutaraldehyde in 0.1 M cacodylate buffer (pH 7.4). The kidneys were diced into 1-mm³ blocks and fixed overnight at 4 °C. The samples were then post-fixed in 1% osmium tetroxide (OsO4) in 0.1 M cacodylate buffer for 2 h at room temperature, dehydrated through graded ethanol (50–100%), passed through propylene oxide, and embedded in epoxy resin. Ultrathin sections (~70 nm) were made using an ultramicrotome (Leica EM UC6, Wetzlar, Germany). The sections were then stained with 4% uranyl acetate for 15 min and 0.5% lead citrate for 5 min. The specimen samples were imaged with a H-7650 transmission electron microscope (TEM) (Hitachi, Tokyo, Japan) at 100 kV. Images were acquired at magnification ranging from ×2000 to ×5000 for detailed observation of podocyte foot processes and slit diaphragm structures. For scanning electron microscopy (SEM), sliced kidney samples were fixed with 3% glutaraldehyde in 0.1 M cacodylate buffer (pH 7.4) and post-fixed 1% OsO_4_, the samples were dehydrated through a graded ethanol series and immersed in t-butyl alcohol for freeze-drying. Specimens in the alcohol were then frozen in 4 °C, and dried with a vacuum evaporator (ID-2, Eiko, Mito, Japan) for 1 h. The specimens were coated with gold vapor to a thickness of approximately 10 nm using a JFC-1600 sputter coater (JEOL, Tokyo, Japan). The specimens were then observed using a JSM-5200 SEM (JEOL, Tokyo, Japan) at an accelerating voltage of 15 kV. Quantitative analysis, including measurements of podocyte foot process width and slit diaphragm length, was performed using ImageJ software (version 1.54m, NIH). A minimum of six glomeruli per sample from at least five mice per group were analyzed to ensure statistical robustness.

### 4.8. Cell Culture

The human kidney-derived podocyte cell line PODO/TERT256 (Evercyte, Vienna, Austria, CHT-033-0256) was used in this study. We have signed MTAs with companies. PODO/TERT256 cells are known to maintain physiological properties of primary cells and can be cultured extensively [60]. The cells were cultured at 37 °C in a 5% CO_2_ environment using cell culture dishes pre-coated with human collagen I. The cells were fed with MCDB 131 medium (Thermo Fisher Scientific, Waltham, MA, USA) which has the same components as the recommended medium, supplemented with GlutaMAX-I (1.6 mM) (Thermo Fisher Scientific, Waltham, MA, USA), Bovine Brain Extract (9.6 μg/mL) (Lonza, Basel, Switzerland), hEGF (8 ng/mL) (Sigma-Aldrich, St. Louis, MO, USA), Hydrocortisone (20 ng/mL) (Sigma-Aldrich, St. Louis, MO, USA), G418 (100 µg/mL) (Fujifilm Wako, Osaka, Japan), and 20% fetal bovine serum. The number of podocytes was determined based on the specific requirements of each experiment. After 7–10 days of differentiation, cells were subjected to experimental treatment. Treatment with each drug was carried out under serum-free conditions.

### 4.9. RNA Isolation and RT-PCR

RNA isolation was conducted following a previously described protocol [59]. Gene-specific primers were synthesized by Sigma-Aldrich (St. Louis, MO, USA). The sequence for the human primers employed were as follows: Two individually designed primer for *Sigmar1* (NM_005866.4) were used for this study: (1), forward and reverse primers: 5′-AGCGCGAAGAGATAGC-3′, and 5′-AGCATAGGAGCGAAGAGT-3′ (509 bp) [61]; (2), 5′- GGGAGACGGTAGTACACGG-3′, and 5′-AGGAGCGAAGAGTATAGAAGAGG-3′ (171 bp) respectively [50]; *β-actin* (NM_001101.5, 250 bp) forward and reverse primers: 5′-CATGTACGTTGCTATCCAGGC-3′, and 5′-CTCCTTAATGTCACGCACGAT-3′, respectively [62]. To confirm primer specificity, representative gel images of PCR products were captured. PCR was conducted utilizing KOD-Plus-Ver.2 (Toyobo Co., Ltd., Osaka, Japan) as previously delineated [59]. PCR products were visualized using GelRedTM (Fujifilm Wako, Osaka, Japan) staining and separated on 2% agarose gels.

### 4.10. Morphological Analysis, Immunocytochemistry, and Albumin Permeability Assay of Podocytes

Morphological analysis of podocytes were conducted following previously described methods [63]. PODO/TERT256 cells from each experimental group were fixed with 4% formaldehyde and blocked by incubation with 1% bovine serum albumin (BSA) in PBS. For cytomorphological analysis, fixed cells were stained with rhodamine-phalloidin reagent (Abcam, Cambridge, UK) according to the manufacturer’s protocol. For immunocytochemistry, the cells were incubated with primary antibodies, specifically anti-nephrin antibody (1:200) (SC-376522, Santa Cruz, CA, USA) and anti-Sigmar1 antibody (1:200) (15168-1-AP, Proteintech, Rosemont, IL, USA) in PBS (1% BSA) at 4 °C for overnight. Random cell images were captured using a confocal laser scanning microscope (LSM980, Carl Zeiss Microscopy, Jena, Germany) at the appropriate excitation/emission wavelengths. The intensity of rhodamine-phalloidin staining was quantified by a computer image analysis system (Image J, version 1.54m, NIH). Differentiated podocytes were used to assess albumin permeability by quantifying the amount of BSA that crossed the podocyte monolayer following modified methods previously described [24,25,26]. Briefly, the upper layer was filled with serum-free MCDB 131 medium, and the lower layer was filled with MCDB 131 medium containing 50 mg/mL BSA. The medium was collected from the upper layer at different time points (1, 2, and 4 h). The BSA concentration was measured using the BCA method. Infinite M200 microplate reader (Tecan Group Ltd., Männedorf, Switzerland) was used to measure BCA absorbance at 562 nm.

### 4.11. Measurement of Cell Viability and Cytotoxicity

Podocytes were cultured in 96-well plates and maintained in culture medium. Cell viability was assessed using a colorimetric MTT assay kit (Cell count kit-8; Dojindo, Kumamoto, Japan). The cell viability depicted in Figure 2A,F and Figure 3B is presented as a relative value (%) of the absorbance obtained in each experiment, normalized to the average value of the control condition. To assess podocytes cytotoxicity, we employed a colorimetric lactate dehydrogenase (LDH) activity assay kit (Cytotoxicity LDH Assay Kit-WST, Dojindo, Kumamoto, Japan) following the manufacturer’s protocol. Cytotoxicity data, as presented in Figure 2B,H and Figure 3C, were calculated in accordance with the manufacturer’s instructions. Measurements were conducted 48 h after treatment with 0.3 µg/mL ADR using absorbance at 450 nm (MTT assay) or 490 nm (LDH activity) wavelengths on a Multiskan JX plate reader (model 353, Thermo Fisher Scientific, Waltham, MA, USA).

### 4.12. Statistical Analysis

Statistical analyses were performed utilizing GraphPad Prism software (version 9, GraphPad software). The results were presented as mean ± standard error of the mean (SEM). The 50% inhibition concentration (IC_50_) of SA4503 was calculated in GraphPad Prism (version 9, GraphPad software, San Diego, CA, USA), using the sigmoidal dose-response equation “log[inhibitor] vs. response − variable slope”, defined as:

Y = Bottom + (Top − Bottom)/(1 + 10^(LogIC50–X)×Hillslope^)

where “Top” represents the maximal response, “Bottom” represents the maximally inhibited response, and “Hillslope” represents the steepness of the curve. To evaluate the statistical significance between means, we applied Student’s *t*-test following the verification of variance equality via an F-test. A *p*-value of less than 0.05 was deemed statistically significant. For multiple comparisons, one-way analysis of variance (ANOVA) was utilized, followed by Tukey’s post hoc test. To ensure the reproducibility of the results, the experiments were conducted a minimum of three times.

## 5. Conclusions

These results suggest that activation of glomerular Sigmar1 by SA4503 provides protective effects against ADR-induced glomerular injury. Regarding the mechanism of SA4503’s renal glomerular protective effect, in addition to ER stress and anti-inflammatory effects, suppression of nephrin degradation via Sigmar1 activation may be partially involved. Based on these findings, it is suggested that Sigmar1 may be a novel therapeutic target in the treatment of glomerular diseases, including nephritic syndromes. However, this study examined the effects in cultured human podocytes and rodents, and it is expected in the future that animal models similar to clinical conditions and clinical trials will be conducted to examine the long-term effects of SA4503 on nephrotic syndrome.

## Data Availability

The data are contained within the article.
